# The impact of a high‐definition multileaf collimator for spine SBRT

**DOI:** 10.1002/acm2.12197

**Published:** 2017-09-27

**Authors:** Kelly C. Younge, John R. Kuchta, Justin K. Mikell, Benjamin Rosen, Jeremy S. Bredfeldt, Martha M. Matuszak

**Affiliations:** ^1^ Department of Radiation Oncology University of Michigan Health System Ann Arbor MI USA

**Keywords:** complexity, deliverability, HD‐MLC, spine SBRT

## Abstract

**Purpose:**

Advanced radiotherapy delivery systems designed for high‐dose, high‐precision treatments often come equipped with high‐definition multi‐leaf collimators (HD‐MLC) aimed at more finely shaping radiation dose to the target. In this work, we study the effect of a high definition MLC on spine stereotactic body radiation therapy (SBRT) treatment plan quality and plan deliverability.

**Methods and Materials:**

Seventeen spine SBRT cases were planned with VMAT using a standard definition MLC (M120), HD‐MLC, and HD‐MLC with an added objective to reduce monitor units (MU). M120 plans were converted into plans deliverable on an HD‐MLC using in‐house software. Plan quality and plan deliverability as measured by portal dosimetry were compared among the three types of plans.

**Results:**

Only minor differences were noted in plan quality between the M120 and HD‐MLC plans. Plans generated with the HD‐MLC tended to have better spinal cord sparing (3% reduction in maximum cord dose). HD‐MLC plans on average had 12% more MU and 55% greater modulation complexity as defined by an in‐house metric. HD‐MLC plans also had significantly degraded deliverability. Of the VMAT arcs measured, 94% had lower gamma passing metrics when using the HD‐MLC.

**Conclusion:**

Modest improvements in plan quality were noted when switching from M120 to HD‐MLC at the expense of significantly less accurate deliverability in some cases.

## 1 INTRODUCTION

The TrueBeam STx and the EDGE linear accelerator from Varian Medical Systems (Palo Alto, CA, USA) are equipped with a number of features designed to facilitate the high‐precision, high‐accuracy radiation treatment of small targets near critical structures both intra‐ and extracranially.^1^ One such feature is the high‐definition multileaf collimator (HD‐MLC), which uses 32 central 2.5 mm‐width leaves and 28 outer 5 mm‐width leaves on each MLC bank (widths projected to isocenter). The HD‐MLC provides essentially twice the resolution along the axis perpendicular to the leaf travel compared to the standard Varian Millennium MLC (M120) which uses 40 central 5 mm‐width leaves and 20 outer 10 mm‐width leaves in each bank.

For inversely optimized treatment plans, the added resolution of the HD‐MLC theoretically increases the ability of the optimizer to produce a highly conformal treatment plan, sparing organs at risk (OARs) while providing adequate target coverage. However, this potential increase in plan quality depends on the ability of the optimizer to efficiently and effectively search the allowed solution space and additionally is fundamentally limited by the physical properties of the photon beam and MLC leaves.

Our department began using Varian's EDGE linear accelerator for stereotactic treatments in January of 2016. Two of the first treatment types to be transferred almost exclusively to the EDGE were spinal stereotactic body radiation therapy (SBRT) and spinal stereotactic radiosurgery (SRS), both delivered with volumetric modulated arc therapy (VMAT). The geometric complexity of these cases, often with irregularly shaped target volumes that may include multiple vertebral bodies and can circumscribe the spinal cord, makes them excellent candidates for treatment on this specialized accelerator.^2^

With the transfer of these patients to the EDGE, a marked increase in plan modulation was observed, visualized both in the MLC‐leaf trajectory sequence in the Eclipse treatment planning system (TPS) as well as quantified by the number of monitor units (MU) and our in‐house VMAT complexity metric.^3^, ^4^ We also observed a sudden increase in plans failing patient‐specific quality assurance with the ArcCHECK (Sun Nuclear Corporation, Melbourne, FL, USA).

Because there is often very little extra time built into the workflow for hypofractionated spine treatments, a failing pretreatment quality assurance measurement can lead to significant disruption in the planned treatment course. The case may either be replanned, which often leads to a delay (or multiple delays) in the patient start date, or the treatment team may elect to proceed with the initial treatment plan and accept the added degree of delivery uncertainty. The goal of this work was to evaluate the change in plan quality and delivery accuracy when switching from the M120 to HD‐MLC, and to test the effect of an added optimization objective aimed at reducing plan modulation on these quality indices.

## 2 METHODS

For this study, 17 previously treated spine SBRT cases employing the M120 were selected for study. A variety of sites (3 C‐spine, 11 T‐spine, and 3 L‐spine) were chosen with geometries representative of that typically seen in our clinic. To ensure consistency in planning and plan quality, all cases chosen were replanned by an expert dosimetrist using the current clinical version of our treatment planning system, Eclipse version 13.6. Each of the cases was inverse planned with VMAT with the Photon Optimizer on a fine (1.25 mm) grid using 2 arcs per our institutional standard for spine SBRT. The final dose calculation was done on a 1 mm grid with Analytical Anisotropic Algorithm (AAA) version 13.6.23. All plans had a prescription dose of 30 Gy delivered in three fractions and used 6 MV‐only beams. All plans were reviewed by a physicist and physician with experience in spine SBRT. Once an acceptable plan was generated (henceforth referred to as the *M120* plan), for each case, three additional versions of the plan were created, as shown in Table 1. Consistent geometry was used across all plans within a given case (i.e., identical arc rotation length, collimator angles and jaw positions).

**Table 1:** Description of plans used in this study

Plan name	Description
*M120*	Original plan using M120 MLC
*M120(HD)*	*M120* plan recreated with HD‐MLC by pairing the MLC leaves
*HD*	*M120* plan reoptimized with identical objectives on HD‐MLC
*HD(MU)*	*HD* plan reoptimized with MU objective to reduce MU to that of the *M120* plan

The *M120(HD)* plan was created by converting the *M120* plan to HD‐MLC by grouping pairs of leaves to identically match the original MLC pattern, and then adjusting the MLC positions outwards (making larger apertures) by 0.7 mm to account for the fact that the dosimetric leaf gap (DLG) for the HD‐MLC is smaller compared to the M120.^5^ The adjustment amount was chosen empirically by comparing the doses in the *M120* plan to the *M120(HD)* plan. Edits to the MLC positions were performed using an in‐house Matlab script (Mathworks, Natick, MA, USA).

The *HD* plan was created by reoptimizing the *M120* plan with the HD‐MLC, using identical optimization objectives. The *HD(MU)* plan was the same as the *HD* plan but had an added MU objective (a penalty on an MU value above a given threshold) to reduce the MU down to that of the *M120* plan.

To evaluate plan quality and modulation complexity, the *M120* plan was compared to the *HD* and *HD(MU)* plans. To evaluate delivery accuracy, we compared the *M120(HD)* plan to the *HD* and *HD(MU)* plans. This approach was taken so that in terms of deliverability, all plans could be compared on an equal footing: using the same beam model, calculation algorithm, and delivery and measurement system. This excluded any potential differences in the accuracy of each workflow step for the M120 MLC versus the HD‐MLC from biasing our results. Additionally, with this approach, small deviations in the dose distribution due to changing MLCs (i.e., converting *M120* to *M120(HD)*) had no impact on the results of the study.

To quantify plan quality, several metrics for the target and nearby OARs were used. Conformity index (CI) and gradient index (GI) were used to quantify dose coverage and falloff between the three types of plans. The conformity index used here was the Paddick index, given by:

(1) $$CI=\frac{TV_{PI}^{2}}{PIV\times TV}$$

where TV_PI_ is the target volume encompassed by the prescription isodose surface, PIV is the prescription isodose surface volume, and TV is the target volume.

The gradient index was defined as

(2) $$GI=\frac{PI_{50\%}}{PIV}$$

where PI_50%_ is the volume encompassed by the 50% dose isodose surface. 6

To quantify plan complexity, we used a complexity metric developed in‐house that analyzes an MU‐weighted average of the leaf‐side perimeter divided by the aperture area.^3^ This metric was designed to quantify plan modulation independent of target size, plan dose, arc length, and MLC type, and is defined as,

(3) $$M=\frac{1}{MU}\sum_{i=1}^{N}MU_{i}\times \frac{y_{i}}{A_{i}},$$

where the sum is over all control point apertures from *i = 1* to *N*,* MU* is the total number of MU in the plan, *MU*_*i*_ is the number of MU delivered through aperture *i*,* A*_*i*_ is the open area of aperture *i*, and *y*_*i*_ is the aperture perimeter excluding the MLC leaf tips.

Our portal dosimetry measurements used the digital megavoltage imager (DMI) on the EDGE linear accelerator and the portal dosimetry application within Eclipse. This DMI was clinically commissioned for 6 MV photons with the Portal Dosimetry Image Prediction (PDIP) algorithm version 13.6.23. For the dose comparisons in this study, we used a locally normalized gamma analysis with 10% dose threshold and no region of interest. Local normalization was used to help highlight potential differences in the deliverability between plans. All measurements were autoaligned to the predicted image by the portal dosimetry software, and our clinical agreement criteria of 4%/1 mm were applied.

In all cases shown below, statistical significance in the comparison between plans was determined using the two‐tailed Student's *t*‐test.

## 3 RESULTS

**Figure 1:**
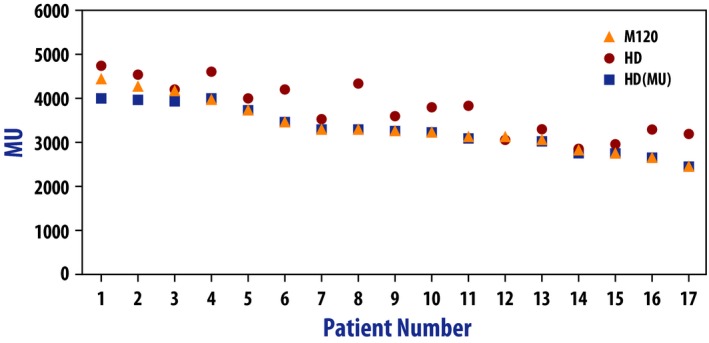
Comparison of MU for the *M120*,*HD*, and *HD(MU)* plans. The graph is ordered from highest MU to lowest MU for the *M120* plan.

**Figure 2:**
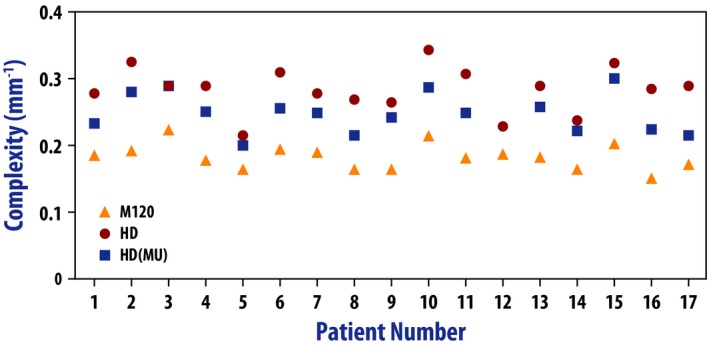
Comparison of modulation complexity for the *M120*,*HD*, and *HD‐MU* plans. The data are ordered identically to Fig. 1.

Figures 1 and 2 illustrate the differences in the MU and modulation complexity for the three types of plans used in this study (*M120*,* HD*, and *HD(MU)*). The cases are sorted by the MU of the *M120* plan. One case (case 12) did not have an *HD(MU)* plan because the MU of the *HD* plan was already lower than that of the *M120* plan.

In the majority of cases, the MU‐objective in the optimizer was able to reduce the MU in the *HD(MU)* plan down to that of the original *M120* plan. The maximum value that can be used in the MU objective is 4000. There were three cases where the *M120* plan had MU >4000, which resulted in the *HD(MU)* plan having fewer MU than the M120 plan (see the left‐most three cases in Fig. 1). The modulation complexity of the *HD* plan was consistently higher than the corresponding *M120* plan. Using the MU objective slightly reduced the modulation complexity in all but one case (case 3), but not to the level of the *M120* plan.

Table 2 summarizes the data of Figs. 1 and 2. Both the MU and the modulation complexity increased significantly between the *M120* and the *HD* plans. The added MU objective reduced both the MU and the complexity, but these values were still significantly higher than those for the *M120* plans.

**Table 2:** Average MU and Complexity values for the 17 test cases

	*M120*	*HD*	*HD(MU)*	*P*‐value		
				*M120* vs *HD*	*M120* vs *HD(MU)*	*HD* vs *HD(MU)*
Average MU	3370 ± 585	3783 ± 613	3315 ± 507	<0.001	0.07	<0.001
Average Complexity (mm^−1^)	0.18 ± 0.02	0.28 ± 0.03	0.25 ± 0.03	<0.001	<0.001	<0.001

**Figure 3:**
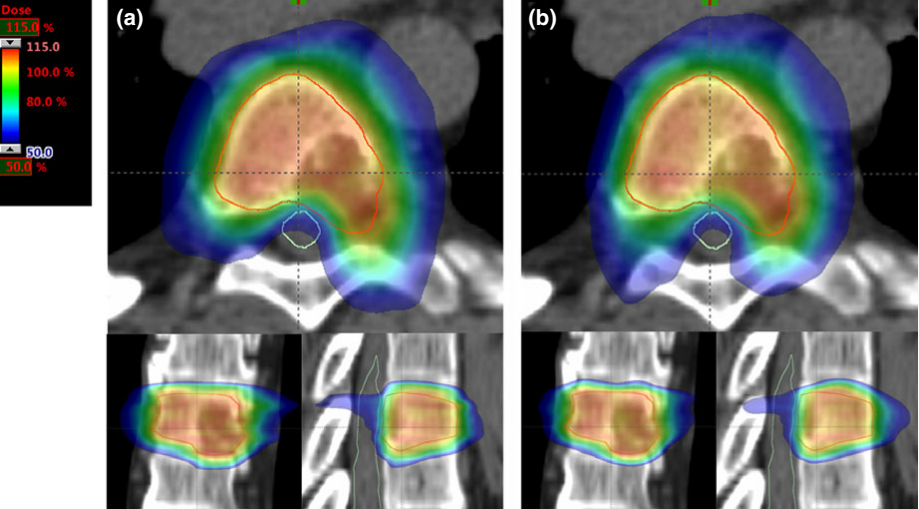
Comparison of two plans with differing gradient indices. The plan on the left (a) has a gradient index of 5.23 and the plan on the right (b) has a gradient index of 5.01. The color wash ends at 50% of the prescription dose.

Table 3 compares dosimetric parameters for the 17 test cases. Some of the comparisons are statistically significantly different, but the absolute difference may not be clinically relevant. The average gradient index in the *HD* plans was 0.19 lower than in the *M120* plans. Figure 3 illustrates the potential difference in dose distributions for this magnitude of change in GI. There is no clear best type of plan according to these metrics.

**Table 3:** Plan quality parameters of *M120* and *HD‐MLC* plans

Quality Metric	*M120*	*HD*	*HD(MU)*	*P*‐Value		
				*M120* vs *HD*	*M120* vs *HD(MU)*	*HD* vs *HD(MU)*
CI	0.72 ± 0.10	0.74 ± 0.10	0.73 ± 0.10	0.01	0.003	0.17
GI	5.08 ± 0.96	4.88 ± 0.96	4.86 ± 0.91	0.01	<0.001	0.43
PTV D98 (Gy)	22.7 ± 5.1	22.3 ± 5.3	22.7 ± 5.1	0.02	0.96	0.01
PTV D90 (Gy)	28.6 ± 3.0	28.6 ± 2.9	28.5 ± 3.0	0.56	0.10	0.40
Spinal Cord 0.1 cc (Gy)	16.6 ± 1.1	16.1 ± 0.9	16.5 ± 0.9	0.01	0.69	0.01

**Figure 4:**
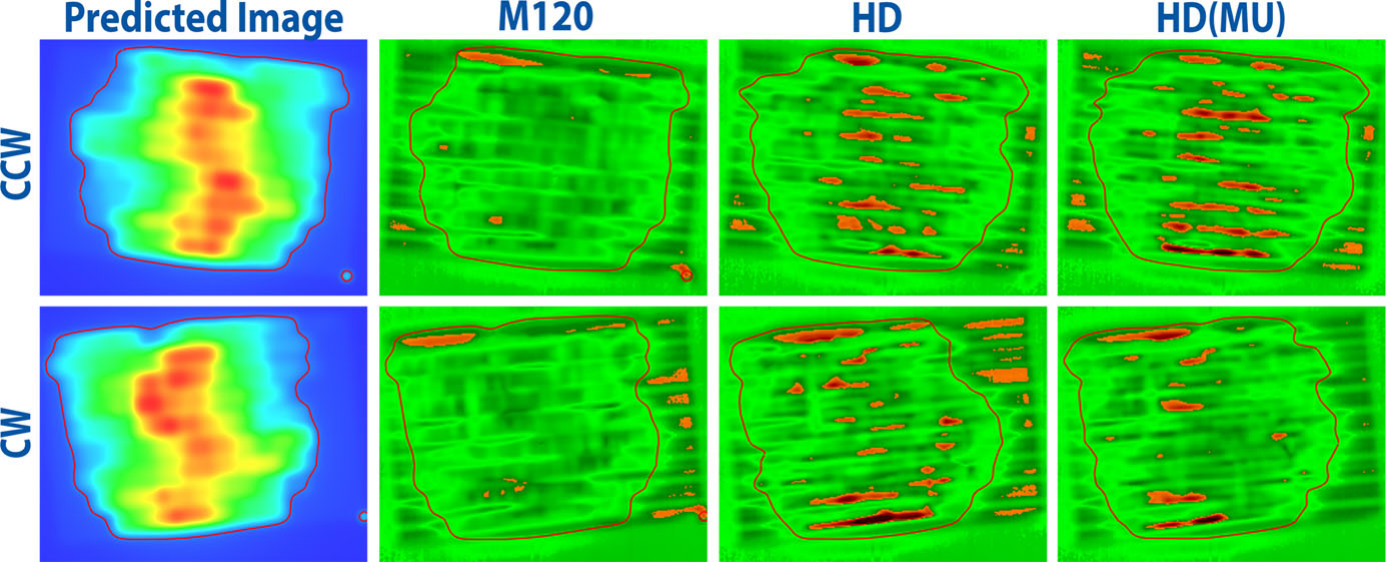
Example portal dosimetry measurement for one case with arcs named CW (clockwise) and CCW (counter clockwise). The predicted images are shown on the left (for the *M120* plan), with gamma maps (4%/1 mm, local normalization) on the right. The 10% dose threshold is outlined in red. Orange pixels indicate failing measurement points (gamma >1).

Figure 4 shows an example portal dosimetry measurement. Only the predicted images for the *M120* plan are shown. The passing percentage is obtained by computing the number of passing measurement points within the outlined area (defined by a 10% dose threshold). Local analysis can cause exaggerated failures in low dose regions; these points are not included in the pass rate percentage.

**Figure 5:**
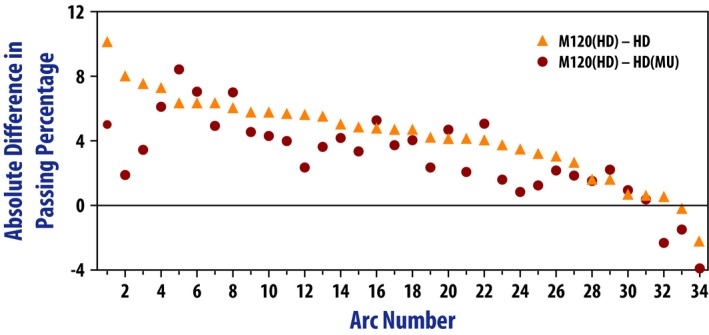
Difference in per arc gamma passing percentages between the *M120(HD)* plans and the *HD* and *HD(MU)* plans. Positive values mean that the *M120(HD)* arc had a higher passing rate than the comparison arc. The graph is ordered from highest difference between M120(HD) and HD to lowest difference between M120(HD) and HD passing percentage.

Figure 5 and Table 4 show the results of the portal dosimetry analysis for the *M120(HD)*,* HD*, and *HD(MU)* plans. These results are per arc (two arcs per plan). For 2 arcs out of the 34 measured, the *HD* and *HD(MU)* plans had higher passing rates than the corresponding *M120(HD)* plan. For one arc, the *M120(HD)* plan was better than *HD* but worse than the *HD(MU)*. For all other arcs, the *M120(HD)* outperformed the other two. All average passing rates were statistically significantly different from each other. For the portal dosimetry measurements, gamma analysis with local normalization was used to avoid artificially raising the passing rates for highly modulated plans. Average global normalization passing rates for the 51 measured plans are also included in Table 4 for reference.

**Table 4:** Summary of portal dosimetry results. A passing arc is defined as a defined as an arc with a gamma pass rate of greater than 95%. A passing plan is defined as a plan where both arcs pass. All measurements are locally normalized unless otherwise stated

	*M120(HD)*	*HD*	*HD(MU)*
Average arc pass rate	99.1 ± 1.0 (*P* < 0.001 vs *HD*)	94.4 ± 2.3 (*P* < 0.001 vs *HD(MU)*)	95.5 ± 2.3 (*P* < 0.001 vs *M120(HD)*)
Percentage of passing arcs	94.1%	41.2%	58.8%
Percentage of passing plans	94.1%	29.4%	52.9%
Percentage of passing plans (using global normalization)	94.1%	78.9%	85.7%

## 4 DISCUSSION

### 4.A Plan quality

Plans generated with the *HD‐MLC* tend to have better spinal cord sparing (about 3% of cord max dose) than the *M120* plans, however, dose falloff and conformity were only marginally better when using the *HD‐MLC*. Though some of the differences are statistically significant, they may not be clinically relevant. The MU and complexity score both increased dramatically in order to achieve these modest gains, and using the MU objective was not effective in reducing the complexity though it worked well to reduce the MU to the desired range. We note that the programming of the MU objective was adjusted in version 13.6 of Eclipse and users of earlier versions may not reproduce the same results as seen here.

### 4.B Deliverability

Portal dosimetry measurements show that the spine plans optimized with the HD‐MLC have significantly degraded deliverability. Using global normalization (last row of Table 4), the passing rates of the plans generated for this study were very similar to the passing rates we observed with clinical plans prior to the initiation of this study. Using local normalization, over two‐thirds of the measured *HD* plans fell below a 95% pass rate threshold. The deliverability was somewhat improved with the added MU objective, but did not match that of the original *M120* plan. This was expected as though the MU were lower for the *HD(MU)* plans, the modulation complexity scores were still high compared to the *M120* plans. We note that all plans used in the comparison of deliverability were calculated using the same beam model and delivered using the same delivery platform. However, because the *HD* and *HD(MU)* plans are more modulated, they are likely to be more sensitive to any weaknesses in the beam model or MLC parameters such as transmission and DLG.

### 4.C Comparison to previous studies

Many other groups have performed planning studies to examine the benefit of the smaller HD‐MLC leaf width. Chae et al found that for artificially contoured spine lesions, the HD‐MLC significantly improved GI but not CI, but that the results more notable for IMRT vs VMAT. The biggest gains were seen for complex target shapes.^7^ Tanyi et al performed a planning study comparing the M120 to HD‐MLC for liver and lung with 3D, IMRT, and VMAT treatment plans and found small differences between the MLC types with the biggest difference being the faster dose falloff with the HD‐MLC.^8^ Dhabaan et al examined the benefit of the HD‐MLC for intracranial radiosurgery planning with dynamic conformal arcs and found that conformity and dose gradients were both improved with the HD‐MLC.^9^

Though there is no question that the HD‐MLC may provide a benefit for certain body sites and certain types of target geometries, there have been very few studies looking at the deliverability impact of the HD‐MLC. Wuu et al used CT‐based polymer gel dosimetry to show that for a 2.5 cc radiosurgery volume treated with IMRT, CI and dose falloff were both improved with the smaller MLC..^10^ Kairn et al did not use the HD‐MLC but studied delivery accuracy for the treatment of spinal metastases using portal dosimetry and radiochromic film.^11^ They found that IMRT plans tended to be more deliverable than dosimetrically similar VMAT plans even though the delivery time was longer. Similar to the present study, the authors were able to improve delivery accuracy by limiting the MU of the plans.

We note that one weakness of this study is that the PDIP algorithm is a standalone algorithm that is configured independently of the AAA algorithm (although it does have some shared beam data), and therefore does not directly verify the dose calculation of AAA.^12^ While our clinical ArcCHECK experience appears to support the findings in this work that deliverability is reduced with the HD‐MLC, the ArcCHECK is a coarse measurement compared to the target size of most of the spine plans studied. Further analysis of these plans could be performed using film to more thoroughly verify the AAA dose calculation strengths and weaknesses.

The solution found by the optimizer for the *M120* plans is part of the solution set available for the *HD* and *HD‐MU* plans, however, the optimizer tends to choose more highly modulated plans. This is partially a result of the fact that the optimizer does not have direct feedback about the modulation in the plan, outside of the number of MU, and therefore is not able to directly limit the modulation.^3^ The MU objective is not a sufficient tool for ensuring the deliverability of plans while at the same time effectively searching for an acceptable dose distribution.

In practice when using the HD‐MLC, the user will not typically have a comparison M120 plan, so it is difficult or impossible to effectively judge the modulation complexity versus the plan quality to find an acceptable solution. Using a complexity metric like the one employed here aids in comparing newly created plans to past plans that have previously been measured for delivery accuracy. Besides analyzing complexity postoptimization, planners need improved tools for limiting modulation when it is not needed in order to ensure an accurate delivery of the dose. One potential solution is to penalize complexity during the optimization in order to help guide the optimizer into the solution space that represents an acceptable compromise between plan quality and deliverability.^3^ Though in this work the HD‐MLC plans exhibit modest gains in the quality of the dose distribution, this improvement will come at a cost in terms of calculation accuracy, delivery accuracy, and delivery efficiency. Additionally, if calculation and delivery accuracy are sacrificed, one cannot be sure that the promised dosimetric gains are in fact realized.

## 5 CONCLUSION

The HD‐MLC theoretically allows improved dose distributions through added degrees of optimization freedom, however, care must be taken during the optimization process to avoid needlessly increasing the level of plan modulation. We have shown that increased plan complexity may lead to increased delivery uncertainty while at the same only modestly improving the dosimetric quality of the plan.

## CONFLICT OF INTEREST

The authors declare that they have no conflicts of interest.
